# Intrathecal Th17-driven inflammation is associated with prolonged post-treatment convalescence for patients with Lyme neuroborreliosis

**DOI:** 10.1038/s41598-023-36709-w

**Published:** 2023-06-15

**Authors:** Paula Gyllemark, Johanna Sjöwall, Pia Forsberg, Jan Ernerudh, Anna J. Henningsson

**Affiliations:** 1 Department of Infectious Diseases, Region Jönköping County, 551 85 Jönköping, Sweden; 2 Division of Inflammation and Infection, Department of Biomedical and Clinical Sciences, Linköping University, Linköping, Sweden; 3 Department of Infectious Diseases, Region Östergötland County, Linköping/Norrköping, Sweden; 4 National Reference Laboratory for Borrelia and Other Tick-Borne Bacteria, Division of Clinical Microbiology, Laboratory Medicine, Region Jönköping County, Jönköping, Sweden

**Keywords:** Immunology, Medical research

## Abstract

Lyme neuroborreliosis (LNB) is associated with increased levels of pro-inflammatory cytokines and chemokines in the cerebrospinal fluid (CSF). Residual symptoms after antibiotic treatment can have deleterious effects on patients and knowledge regarding the pathogenesis linked to prolonged recovery is lacking. In this prospective follow-up study, we investigated the B cell-associated and T helper (Th) cell-associated immune responses in well-characterized patients with LNB and controls. The aims were to assess the kinetics of selected cytokines and chemokines involved in the inflammatory response and to identify potential prognostic markers. We investigated 13 patients with LNB according to a standardized clinical protocol before antibiotic treatment and after 1, 6 and 12 months of follow-up. CSF and blood samples were obtained at baseline and after 1 month. As controls, we used CSF samples from 37 patients who received spinal anesthesia during orthopedic surgery. The CSF samples were analyzed for CXCL10 (Th1-related), CCL22 (Th2-related) and IL-17A, CXCL1 and CCL20 (Th17-related), as well as for the B cell-related cytokines of a proliferation-inducing ligand (APRIL), B cell-activating factor (BAFF) and CXCL13. The CSF levels of all the cytokines and chemokines, with the exception of APRIL, were significantly higher at baseline in patients with LNB compared with controls. All the cytokines and chemokines, except for IL-17A were significantly reduced at 1-month follow-up. Patients with quick recovery (< 1 month, n = 3) had significantly lower levels of CCL20 at baseline and lower levels of IL-17A at 1-month follow-up. Patients with time of recovery > 6 months (n = 7) had significantly higher levels of IL-17A at the one-month follow-up. No other cytokines or chemokines were associated with prolonged recovery. Dominating residual symptoms were fatigue, myalgia, radiculitis and/or arthralgia. In this prospective follow-up study of patients with LNB, we found significantly lower levels of CCL20 in those who recovered rapidly, and increased levels of IL-17A in patients with delayed recovery post-treatment. Our findings indicate persistent Th17-driven inflammation in the CSF, possibly contributing to a longer convalescence, and suggest IL-17A and CCL20 as potential biomarker candidates for patients with LNB.

## Introduction

Lyme neuroborreliosis (LNB), which is the dominant form of disseminated Lyme borreliosis in Europe, entails a nervous system infection caused by spirochetes of the *Borrelia (B.)burgdorferi* sensu lato (s.l.) complex^1^. According to European guidelines, a definitive diagnosis of LNB is based on symptoms in combination with the detection of intrathecal production of *Borrelia*-specific antibodies and mononuclear pleocytosis in the cerebrospinal fluid (CSF)^1^. Despite adequate antibiotic treatment, some patients experience residual symptoms, such as fatigue, head pain, musculoskeletal pain and neurocognitive impairment^2^, and it has been suggested that dysregulation of immune responses contributes to these symptoms^3^. The B cell-related chemokine CXCL13 is clinically useful because it is present at raised levels prior to antibiotic treatment and in rapidly decreasing levels post-treatment^4,5^. However, CXCL13 has not been evaluated as a prognostic marker for patients with LNB, and to date no other markers that predict disease course and outcome have been reported.

Measurements of different cytokines and chemokines in the CSF during the disease course of LNB yield detailed understanding of the disease pathogenesis. Previous studies have suggested that a favorable outcome for patients with LNB is associated with a strong initial T helper (Th) 1-immune response, exemplified by the Th1 signature cytokine interferon (IFN)-γ, followed by a counteracting Th2-immune response, represented by interleukin (IL)-4^3^. The Th1-associated chemokine CXCL10, which is induced by IFN-γ ^6^, is a strong chemoattractant for T-lymphocytes into the CNS of patients with LNB^4,7,8^, although the impact of CXCL10 on the disease course remains unclear.

The chemokine CCL22, is strongly associated with Th2 responses^9^, and in vitro studies have shown that microglia, the resident macrophages in the CNS, produce CCL22 upon stimulation with live *B. burgdorferi* s.l.^10^. However, studies, carried out to date have failed to show a correlation between the levels of CCL22 and a more favorable outcome for patients with LNB^7^. In contrast, the Th17-associated immune response, which is known to be involved in different autoimmune disorders^11^, has shown to be associated with long-lasting symptoms in Lyme arthritis and LNB, in which high levels of the cytokine IL-17A have been observed^12,13^. The chemokines CXCL1 and CCL20, both of which are induced by Th17 cells, have been detected at high levels in the CSF samples of patients with LNB^13^. The B cell-related cytokines, a proliferation-inducing ligand (APRIL) and B cell-activating factor (BAFF) are involved in B-cell development and survival^14^. These cytokines are also involved in inflammatory and immune-mediated diseases^15^, suggesting associations with APRIL, BAFF and clinical outcome in patients with LNB, with higher levels in patients with a longer period of convalescence^13^.

Expanded knowledge of the kinetics of the local inflammatory response in the CSF of patients with LNB would further elucidate the pathogenesis of this disease and could identify biomarkers and future therapeutic targets. In the present study, which involved CSF samples that were collected prospectively from patients with LNB, both at baseline and at a follow-up 1-month post-treatment, we investigated the kinetics of CXCL10 (Th1), CCL22 (Th2) and IL-17A, CXCL1 and CCL20 (Th17) as well as APRIL, BAFF and CXCL13 (B cell-related), and assessed their relationships to disease course, with the aim of identifying potential prognostic markers.

## Patients and methods

### Patients

Adult patients aged ≥ 18 years (n = 60) who were admitted to the Department of Infectious Diseases, County Hospital Ryhov in Jönköping with suspicion of LNB, were recruited prospectively in the period of 2005–2010. A standardized protocol with clinical investigation and informed consent were included at the initial visit at baseline, in addition to lumbar puncture (LP) and blood sampling. The standardized protocol included questions regarding common and more uncommon symptoms associated with Lyme neuroborreliosis and a thorough neurological clinical status (Supplementary file). Only patients who had definite LNB (n = 13) according to the guidelines of the European Federation of the Neurological Societies (EFNS) were included in this study^1^. Thus, these patients had symptoms compatible with LNB, CSF pleocytosis (> 5 × 10^6^ mononuclear cells per litre of CSF), and evidence of intrathecally produced *Borrelia*-specific IgM and/or IgG antibodies. CSF and blood samples were drawn prior to antibiotic treatment, which was initiated directly after the first visit, within one day after the LP. After 1 month, the patients were re-examined according to the same study protocol as was used at baseline and an additional LP together with blood sampling were performed in all but one of the patients with LNB. This patient declined a second LP for personal reasons. All the patients took part in the subsequent clinical follow-up visits at 6 and 12 months. At these visits the patients were examined according to the same study protocol as the visit at baseline and 1-month follow up. All the patients with LNB were treated with oral doxycycline according to Swedish treatment guidelines^16^.

Control subjects (n = 37) were recruited from the Department of Orthopedic Surgery, County Hospital Ryhov, Jönköping. These patients received spinal anesthesia due to hip– or knee replacement surgery between 2016 and 2017. Patients with autoimmune or malignant diseases, diabetes or immunosuppression (through disease or medication affecting the immune system) were excluded. Only those control subjects who had a normal CSF cell count and undetectable *Borrelia*-specific antibodies in the serum and CSF were included.

### Serum and CSF

The CSF cell count, albumin ratio [CSF-albumin/serum (S)-albumin] and total IgG index [(CSF-total IgG/S-total IgG)/(CSF-albumin/S-albumin)]^17^ were determined for the patients with LNB at the Department of Clinical Chemistry, County Hospital Ryhov, Jönköping, both at baseline and at one-month follow up. The total IgG index was determined in all but two of the patients with LNB at one-month follow up. One patient declined a second LP and in one patient only limited CSF sample volumes being available. *Borrelia*-specific IgM and/or IgG antibodies in the serum and CSF were analyzed at the Department of Clinical Microbiology, County Hospital Ryhov, Jönköping, using the Lyme Borreliosis ELISA Kit 2nd Generation (Dako Cytomation A/S, Glostrup, Denmark) during the period of 2005–2008. Intrathecal production of *Borrelia-*specific antibodies was assessed by calculation of the antibody index, as described by Peter^18^, with the modification that total IgG was substituted with *Rubella*-specific IgG. A *Borrelia*-specific antibody index > 2 was interpreted as indicative of intrathecal anti-*Borrelia* antibody production. From 2009 onwards, the IDEA Lyme Neuroborreliosis kit (Dako Cytomation) was used for paired serum and CSF samples, and Enzygnost Lyme link VlsE/IgG and Enzygnost Borrelia Lyme IgM (Siemens Healthcare Diagnostics Products GmbH, Marburg, Germany) were used for serum samples, with the exception that the controls were not analysed with the Enzygnost Borrelia Lyme IgM (Siemens Healthcare Diagnostics Products GmbH, Marburg, Germany). These analyses were performed as part of the diagnostic workup at the time of sampling. Due to limited sample volumes, the albumin ratio and total IgG index were determined in only 27 of the 37 controls. These analyses were performed as part of another study^19^ conducted at the Clinical Neurochemistry Laboratory at Sahlgrenska University Hospital, Mölndal, Sweden. CSF and serum levels of albumin and total IgG were measured on the Cobas C501 instrument (Roche Diagnostics, Penzberg, Germany). All serum and CSF samples were stored at − 80 °C until analyzed.

### Cytokine and chemokine measurements

CSF concentrations of APRIL, BAFF and CXCL13 were analyzed using the Invitrogen Immunoassay Kit (BMS2008 (APRIL); Life Technologies, Carlsbad, CA, USA) and the Quantikine system (DBLYSOB (BAFF) and DCX130 (CXCL13); R&D Systems, Inc., Minneapolis, MN, USA), respectively. CSF concentrations of CXCL10, CCL22, IL-17A, CXCL1 and CCL20 were measured using the Luminex multiple bead technology (MILLIPLEX Human Cytokine/Chemokine Kit, Millipore Corp., Darmstadt, Germany). All analyses were conducted at the Department of Clinical Microbiology, County Hospital Ryhov, Jönköping, according to the manufacturers’ instructions. The analyses were performed on patient samples at baseline and at one-month follow-up and on the CSF from the controls respectively. The minimum detectable concentrations were as follows: CXCL10, 1.1 pg/mL; CCL22, 3.6 pg/mL; IL-17A, 1.6 pg/mL; CXCL1, 2.3 pg/mL; CCL20, 4.9 pg/mL; APRIL, 0.8 ng/mL; BAFF, 19 pg/mL; and CXCL13, 3.6 pg/mL. Values under the detection limit, for all the cytokines and chemokines, but BAFF, were assigned half the value of the lowest point of the standard curve. For BAFF, the lowest point of the standard curve was negative and consequently, the second-lowest point of the standard curve was used instead. IL-17A and CCL20 could not be detected by the Luminex methodology, they were instead analyzed using the proximity extension assay (PEA) methodology (Inflammation panel; conducted by the Clinical Biomarkers Facility, Science for Life Laboratory, Uppsala University, Uppsala, Sweden). IL-17A and CCL20 levels are presented on a relative log2 scale as arbitrary normalized protein eXpression (NPX) units.

### Tick-borne pathogen-specific PCR measurements in serum and CSF samples

The patients with LNB were also investigated by PCR for other tick-borne pathogens in CSF (n = 12) and serum (n = 13) samples using PCR. One CSF sample was not analyzed due to limited sample volume. The investigated pathogens were: *Borrelia* spp. (including *B. miyamotoi*); *Anaplasma phagocytophilum*; *Rickettsia* spp; *Neoehrlichia mikurensis*; tick-borne encephalitis virus; and *Babesia* spp (Supplementary file).

### Statistical analysis

For statistical analysis, the SPSS ver. 27 software was used. The Mann–Whitney U-test was used for comparisons of continuous data and Fisher’s exact test was used for categorical variables. For comparisons of paired continuous data, the Wilcoxon signed-rank test was used. Two-tailed p-values < 0.05 were considered significant.

### Ethical approval

Written informed consent was obtained from all the patients**.** The study conforms to the World Medical Association Declaration of Helsinki and was approved by the Regional Ethical Review Board of Linköping University, Linköping, Sweden (M106-04, 2011/65-32, 2015/192-32, 2018/388-32, 2019-02449).

## Results

### Patients’ characteristics

The characteristics of the patients and controls are presented in Table 1. The patients with LNB were significantly younger than the controls (p < 0.001), while the gender distribution did not differ between the two groups (p = 1.000). There were no significant differences between men and women regarding any of the clinical or laboratory variables (data not shown). The duration of symptoms before treatment ranged in the LNB group from 0.9 to 8 weeks. Table 2 presents the clinical parameters for the patients with LNB at baseline and at 1, 6 and 12 months of follow-up. The predominant symptoms at baseline were radiculitis and neck pain, followed by myalgia and/or arthralgia, head pain and fatigue. Six patients had facial nerve palsy, which was the only cranial nerve palsy noted in this study. Many of the symptoms had resolved within the first month after initiation of treatment. Seven patients had residual symptoms noted at the 6-month follow-up. Radiculitis, myalgia and/or arthralgia and facial nerve palsy remained present in four patients even at the 12-month follow-up.

**Table 1:** Characteristics of the patients with LNB and the controls.

	Patients	Controls	p-value
	n = 13	n = 37	
Women, n (%)	5 (38)	22 (59)	1.0
Median age years (range)	52 (25–69)	73 (52–84)	**< 0.001**
Median symptom duration before LP, weeks (range)	4.0 (0.9–8.0)	n/a	n/a
Increased *Borrelia-specific* AI, n (%)	13 (100)	0 (0)	n/a
*Borrelia-specific* IgG antibodies in serum, n (%)	10 (77)	0 (0)	n/a
CSF pleocytosis median × 106/L (range)	132 (14–1030)	1 (0–4)	** < 0.001**

Significant values are in [bold].

n, number; LP, lumbar puncture; n/a, not applicable; AI, antibody index; CSF, cerebrospinal fluid; Pleocytosis, is defined as > 5 × 10^6^ mononuclear cells per litre of CSF.

**Table 2:** Clinical characteristics of patients with LNB at baseline and at follow-up.

	Baseline	Follow up at	Follow up at	Follow up at
		1 month	6 months	12 months
	n = 13	n = 13	n = 13	n = 13
Radiculitis, n (%)	9 (69)	3 (23)	2 (15)^a^	1 (7.7)^d^
Neck pain, n (%)	9 (69)	0 (0)	0 (0)	0 (0)
Myalgia -and/or arthralgia, n (%)	8 (62)	3 (23)	3 (23)	3 (23)
Fatigue, n (%)	7 (54)	5 (38)	4 (31)^b^	0
Headache, n (%)	7 (54)	1 (7.7)	1 (7.7)^c^	0 (0)
Facial nerve palsy, n (%)	6 (46)	4 (31)	1 (7.7)	1 (7.7)
Fever, n (%)	4 (36)	0 (0)	0 (0)	0 (0)
Vertigo, n (%)	0 (0)	0 (0)	0 (0)	0 (0)
Concentration difficulties, n (%)	0 (0)	0 (0)	0 (0)	0 (0)

n, number; ^a^, one patient with radiculitis also had fatigue and headache and one patient with radiculitis had myalgia and/or arthralgia; ^b^, one patient with fatigue also had myalgia and/or arthralgia; ^c^, the one patient with headache also had fatigue; ^d^, the patient with radiculitis also had myalgia and/or arthralgia.

### The levels of most cytokines and chemokines are increased post-treatment in the CSF of patients with LNB and decrease rapidly after treatment

**Figure 1:**
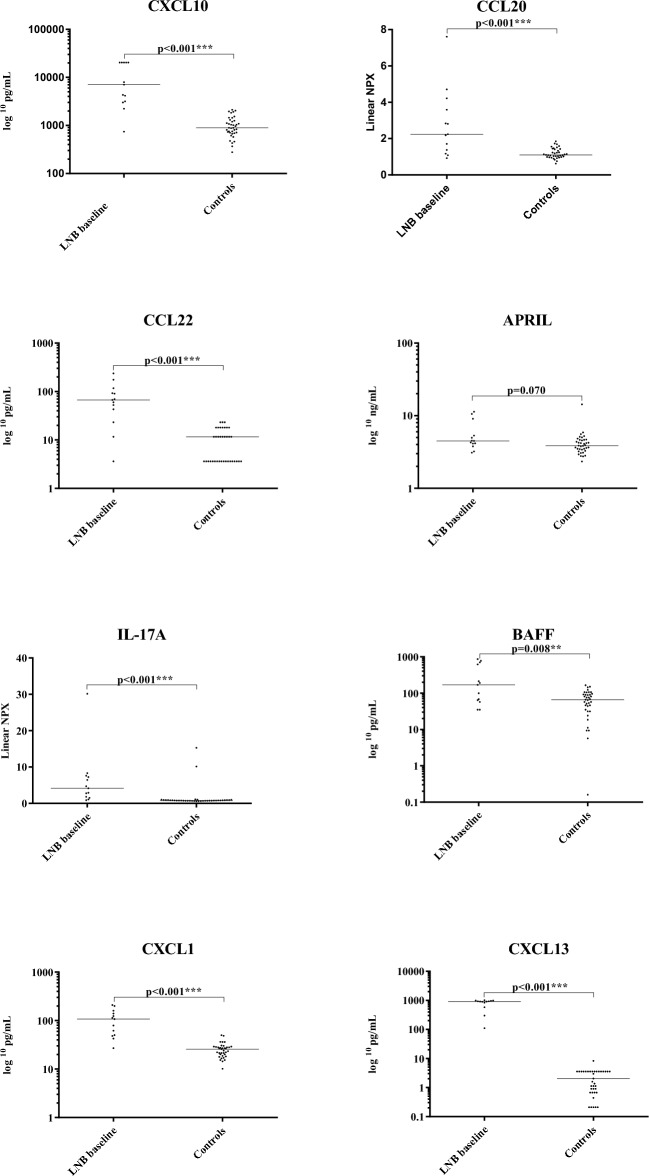
Levels of cytokines/chemokines in CSF of the patients with LNB at baseline and the controls. Every dot represents one patient sample. Horizontal bars represent the median cytokine and chemokine levels.

**Figure 2:**
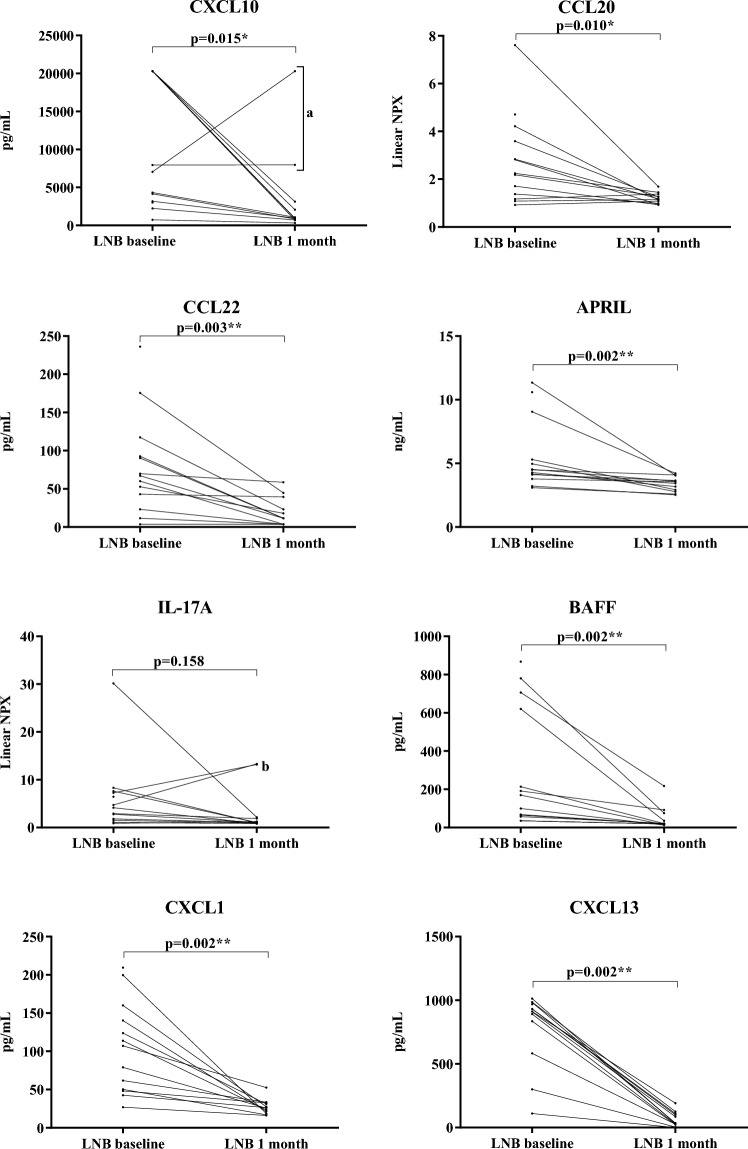
Paired levels of cytokines/chemokines in CSF at baseline and 1-month follow up of the patients with LNB. One of the patients with LNB declined a second LP for personal reasons. (**a**) Two patients with higher levels of CXCL10 after treatment, as compared with their levels at baseline, for whom the positive therapeutic outcome was prolonged (> 6 months) in the patient with almost identical levels of CXCL10 at baseline and at 1-month follow up. (**b**) Three patients with higher levels of IL-17A after treatment, as compared with their levels at baseline, for whom recovery exceeded 6 months.

The patients with LNB had significantly higher CSF levels of the Th1-, Th2- and Th17-associated cytokines and chemokines at baseline compared with the controls (Table 3, Fig. 1). Furthermore, the levels of the B cell-associated CXCL13 and BAFF were higher in the patients with LNB, while differences regarding the levels of APRIL did not reach the level of statistical significance. The levels of most of the tested cytokines and chemokines were significantly reduced in the follow-up samples obtained 1 month after initiation of treatment (Table 3, Fig. 2). An exception to this was the level of IL-17A, the decrease of which was not statistically significant (p = 0.160); moreover, after 1 month, the levels of IL-17A in the patients with LNB were still higher than in the controls (p < 0.001). For CXCL13, there was a statistically significant decrease at follow-up, although the levels were still higher in the patients than in the controls (both p < 0.001). In contrast, the levels of APRIL were slightly increased in the controls compared with the patients with LNB at the 1-month follow-up (p = 0.034).

**Table 3:** Parameters and cytokines/chemokines in the CSF samples of patients with LNB and controls.

	A: Baseline	B: Follow up at	C: Controls	A vs B	A vs C	B vs C
		1 month				
	n = 13	n = 12	n = 37	p-value	p-value	p-value
CSF pleocytosis^a^	140 (14–1000)	13 (2–45)	1 (0–4)	**0.002**	< **0.001**	< **0.001**
Albumin ratio^a^	12 (7–54)	6.3 (4.6–15)	5.7 (4,1–13)^b^	**0.002**	< **0.001**	0.200
Total IgG index^a^	0.7 (0.4–1.2)	0.6 (0.5–0.9)^c^	0.4 (0.4–0.5)^b^	**0.009**	< **0.001**	< **0.001**
CXCL10 (pg/mL)^d^	7100 (3100–20 000)	970 (750–2900)	890 (710–1300)	**0.015**	< **0.001**	0.340
CCL22 (pg(mL)^d^	67 (33–105)	12 (3.6–35)	12 (3.6–18)	**0.003**	< **0.001**	0.150
IL-17A (NPX)^d^	4.1 (1.7–7.4)	1.1 (1.0–2.1)	0.8 (0.7–0.9)	0.160	< **0.001**	< **0.001**
CXCL1 (pg/mL)^d^	110 (49–150)	26 (19–32)	26 (19–29)	**0.002**	< **0.001**	0.680
CCL20 (NPX)^d^	2.2 (1.3–3.9)	1.2 (1.0–1.4)	1.1 (1.0–1.4)	**0.010**	< **0.001**	0.630
APRIL (ng/mL)^d^	4.5 (4.0–7.2)	3.5 (2.8–4.0)	3.9 (3.3–4.5)	**0.002**	0.070	**0.034**
BAFF (pg/mL)^d^	170 (61–660)	19 (18–65)	66 (33–94)	**0.002**	**0.008**	0.053
CXCL13 (pg/mL)^d^	910 (710–980)	34 (24–110)	2.1 (0.7–3.6)	**0.002**	< **0.001**	** < 0.001**

Significant values are in [bold].

n, number; CSF, cerebrospinal fluid; pleocytosis, is defined as > 5 × 10^6^ mononuclear cells per litre of CSF; ^a^median, range; ^b^, data missing for 10 controls; Total IgG index, [(CSF-total IgG/S-total IgG)/(CSF-albumin/S-albumin)]; ^c^, data missing for one patient due to limited volume of CSF; ^d^median, interquartile range; NPX, Normalized Protein eXpression.

### Cytokine and chemokine levels in relation to clinical characteristics

Patients aged > 50 years (n = 5) had significantly higher levels of APRIL at the 1-month follow-up (p = 0.003), as compared with younger patients. Regarding the clinical features, patients with radiculitis (n = 9) had significantly higher BAFF levels at baseline (p = 0.034), and patients with arthralgia and/or myalgia (n = 8) had significantly higher BAFF levels at the 1-month follow-up (p = 0.020) as compared with patients without these symptoms. Patients with a short duration of symptoms (≤ 2 weeks, n = 5) at baseline, had significantly higher levels of CXCL1 (p = 0.030) compared with patients having a longer duration of symptoms. No other significant associations were detected between the cytokine or chemokine levels and the clinical characteristics (data not shown).

### Cytokine and chemokine levels in relation to disease course

In both the patients with rapid recovery (< 1 month, n = 3; Table 4) and with recovery < 6 months (n = 6; Table 5), the levels of the cytokine IL-17A were significantly lower at the 1-month follow-up (p = 0.036 and p = 0.010 respectively), as compared to patients with recovery times > 1 month and ≥ 6 months, respectively. Patients with rapid recovery (< 1 month) also had significantly lower levels of the Th17-associated chemokine CCL20 at baseline (p = 0.049). None of the other tested cytokines or chemokines differed significantly in relation to disease course.

**Table 4:** Parameters, cytokines and chemokines in CSF samples of patients with LNB in relation to short recovery.

	Recovery < 1 month	Recovery > 1 month	p-value
	n = 3	n = 10 (n = 9 LP no. 2)	
(1) CSF pleocytosis^a^	62 (26–174)	160 (14–1000)	0.570
(2) CSF pleocytosis^a^	8.0 (2.0–18)	14 (2.0–45)	0.210
(1) Albumin ratio^a^	12 (11–12)	15 (7.0–54)	0.570
(2) Albumin ratio^a^	6.1 (4.6–9.8)	6.5 (5.1–15)	0.600
(1) Total IgG index^a^	0.69 (0.41–0.79)	0.78 (0.50–1.2)	0.160
(2) Total IgG index^a^	0.57 (0.49–0.64)	0.71 (0.45–0.87)^b^	0.380
(1) CXCL10 (pg/mL)^c^	4300 (740–20,000)	7500 (2200–20,000)	0.690
(2) CXCL10 (pg/mL)^c^	700 (330–1100)	980 (710–20,000)	0.150
(1) CCL22 (pg/mL)^c^	60 (23–90)	69 (3.6–240)	0.690
(2) CCL22 (pg/mL)^c^	3.6 (3.6–12)	18 (3.6–59)	0.150
(1) IL-17A (NPX)^c^	1.5 (1.1–8.3)	4.4 (0.90–30)	0.570
(2) IL-17A (NPX)^c^	0.96 (0.83–0.98)	4.4 (0.90–13)	**0.036**
(1) CXCL1 (pg/mL)^c^	42 (27–200)	110 (48–210)	0.370
(2) CXCL1 (pg/mL)^c^	19 (16–26)	28 (17–53)	0.150
(1) CCL20 (NPX)^c^	1.1 (0.92–2.2)	2.8 (1.2–7.6)	**0.049**
(2) CCL20 (NPX)^c^	1.2 (1.1–1.3)	1.2 (0.93–1.7)	1.000
(1) APRIL (ng/mL)^c^	4.1 (3.1–5.0)	4.5 (3.2–11)	0.290
(2) APRIL (ng/mL)^c^	2.8 (2.6–3.7)	3.6 (2.5–4.2)	0.370
(1) BAFF (pg/mL)^c^	57 (35–170)	200 (35–870)	0.110
(2) BAFF (pg/mL)^c^	18 (15–22)	20 (13–220)	0.370
(1) CXCL13 (pg/mL)^c^	890 (300–1010)	920 (110–990)	0.940
(2) CXCL13 (pg/mL)^c^	28 (4.1–32)	86 (0.21–190)	0.280

Significant values are in [bold].

(1) At baseline; (2) at 1-month follow up; n, number; LP, lumbar puncture; CSF, cerebrospinal fluid; pleocytosis, is defined as > 5 × 10^6^ mononuclear cells per litre of CSF; Albumin ratio, CSF-albumin/S-albumin; Total IgG index, [(CSF-total IgG/S-total IgG)/(CSF-albumin/Salbumin)]; ^a^, Median, range; ^b^, data missing for one patient due to limited volume of CSF; ^c^, Median, interquartile range; NPX, Normalized Protein eXpression.

**Table 5:** Parameters, cytokines and chemokines in CSF in LNB patients in relation to long recovery.

	Recovery < 6 months	Recovery > 6 months	p-value
	n = 6 (n = 5 LP no. 2)	n = 7	
(1) CSF pleocytosis^a^	120 (18–420)	140 (14–1000)	0.950
(2) CSF pleocytosis^a^	8.0 (2.0–45)	14 (9.0–38)	0.340
(1) Albumin ratio^a^	12 (7.0–49)	12 (7.4–54)	0.950
(2) Albumin ratio^a^	6.1 (4.6–15)	6.5 (5.1–14)	0.880
(1) Total IgG index^a^	0.63 (0.41–1.0)	0.83 (0.55–1.2)	0.230
(2) Total IgG index^a^	0.57 (0.45–0.87)	0.71 (0.53–0.82)^b^	0.430
(1) CXCL10 (pg/mL)^c^	5700 (740–20,000)	7900 (2200–20,000)	0.730
(2) CXCL10 (pg/mL)^c^	900 (330–20,000)	980 (710–8000)	0.530
(1) CCL22 (pg/mL)^c^	75 (3.6–240)	67 (12–120)	0.840
(2) CCL22 (pg/mL)^c^	3.6 (3.6–45)	18 (3.6–59)	0.200
(1) IL-17A (NPX)^c^	3.4 (1.1–8.3)	4.7 (0.89–30)	0.630
(2) IL-17A (NPX)^c^	0.96 (0.83–1.1)	1.9 (1.0–13)	**0.010**
(1) CXCL1 (pg/mL)^c^	110 (27–210)	79 (48–160)	0.950
(2) CXCL1 (pg/mL)^c^	23 (16–53)	28 (17–34)	0.530
(1) CCL20 (NPX)^c^	2.5 (0.92–4.7)	2.2 (1.2–7.6)	0.840
(2) CCL20 (NPX)^c^	1.2 (1.1–1.3)	1.1 (0.93–1.7)	0.880
(1) APRIL (ng/mL)^c^	4.3 (3.1–11)	4.5 (3.8–11)	0.450
(2) APRIL (ng/mL)^c^	2.8 (2.5–3.7)	3.6 (2.9–4.2)	0.150
(1) BAFF (pg/mL)^c^	190 (35–870)	99 (35–780)	0.730
(2) BAFF (pg/mL)^c^	20 (15–220)	18 (13–92)	0.880
(1) CXCL13 (pg/mL)^c^	930 (110–1000)	910 (580–980)	0.950
(2) CXCL13 (pg/mL)^c^	28 (0.21–130)	86 (23–190)	0.270

Significant values are in [bold].

(1) At baseline; (2) at 1-month follow up; n, number; LP, lumbar puncture; CSF, cerebrospinal fluid; Pleocytosis is defined as > 5 × 10^6^/mononuclear cells per litre of CSF; CSF; Albumin ratio, CSF-albumin/S-albumin; Total IgG index, [(CSF-total IgG/S-total IgG)/(CSF-albumin/S-albumin)]; ^a^Median, range; ^b^, data missing for one patient due to limited volume of CSF; ^c^, Median, interquartile range; NPX, Normalized Protein eXpression.

### CSF parameters in relation to clinical characteristics and disease course

Patients with a normal total IgG index (< 0.63) at baseline (n = 5) had experienced a significantly shorter symptom duration before treatment (p = 0.030, data not shown), as compared with patients who had an increased total IgG index (n = 8). Patients with head pain and/or neck pain had a significantly higher baseline albumin ratio (p = 0.030, data not shown) compared with patients without these symptoms. There were no significant differences between patients who recovered within 6 months and patients with prolonged (> 6 months) recovery regarding CSF pleocytosis (p = 0.950), albumin ratio (p = 0.950) or total IgG index (p = 0.230) (Table 5).

### PCR measurements of CSF and serum

The PCR assays of the CSF and serum samples were all negative for targeted tick-borne pathogens.

## Discussion

In this study, we describe the involvement of both Th17-related and B cell-related immune responses in patients with LNB, *i.e*., beyond the previously well-described Th1- and Th2-associated immune responses. Our prospective and well-characterized patient cohort with standardized clinical follow-up visits and a second LP provides insights into the kinetics of cytokine and chemokine expression in the CSF and their significance for the disease course.

Half of the patients with LNB had a prolonged recovery period (> 6 months), which was dominated by symptoms such as facial palsy, radiculitis, myalgia and arthralgia, i.e. well-known residual symptoms as presented in the systematic review of Dersch et al.^2^. We found, not only the presence of Th17-related cytokines and chemokines in the CSF samples of patients with LNB, in similarity to previous studies^7,13,20–23^, but also increased baseline levels of IL-17A, CXCL1 and CCL20, as compared with the controls. In contrast to most other cytokines and chemokines that showed decreased levels at the 1-month follow-up, IL-17A was distinguished by sustained high levels in the patients compared with the controls. Moreover, Th17-associated IL-17A and CCL20 were the only cytokines/chemokines that were associated with time of recovery; the levels of IL-17A at the 1-month follow-up were higher in patients with a delayed (> 6 months) recovery and lower levels of the Th17-induced chemokine CCL20 were associated with a short time to recovery (< 1 month). Interestingly, the Th17 type of immunity has been implicated in other neuroinflammatory conditions, such as multiple sclerosis (MS) and its experimental animal model of autoimmune encephalomyelitis (EAE)^24^. In EAE, Th17 cells are important drivers of CNS inflammation through its signature cytokine IL-17A^11^. In addition, the Th17-recruiting chemokine CCL20 is expressed in the choroid plexus and, thus attracts Th17 cells to the CNS by binding its receptor CCR6, which is expressed on Th17 cells^24^. In LNB, increased CSF levels of IL-17A have previously been described^7,20^. Henningsson et al.^7^ found increased CSF levels of IL-17A in half of the investigated patients with confirmed LNB in a retrospective cohort. Furthermore, a high percentage of these patients reported fatigue, although no correlations were noted between the baseline level of IL-17A and the duration of symptoms. In line with our findings, Pietikäinen et al.^22^ have reported significantly increased levels of CXCL1 and IL-17 in the CSF both before and after treatment in 23 patients with LNB, as compared to non-LNB controls; however, that study did not include information on disease course. In another study^25^, factors associated with a subclinical course of *Borrelia* exposure were investigated in asymptomatic, seropositive individuals compared to symptomatic individuals who had been previously diagnosed with LNB. The main factors linked to a subclinical course included lower levels of secretion of IL-17A and CCL20 by *Borrelia*-stimulated mononuclear cells. Thus, a propensity for a restricted Th17 response may contribute to a subclinical course after *Borrelia* exposure, which is in line with our finding that an uncontrolled Th17 response may instead contribute to a delayed disease course in LNB. Based on the aggregated findings from previous studies and our present study, we hypothesize that IL-17A and CCL20 are useful as prognostic markers of disease course in patients with LNB post-treatment. We therefore suggest that a second LP should be considered for patients with residual symptoms, in order to assess ongoing Th17-driven inflammation. Even though our findings need to be confirmed in larger studies, they lend support to the notion that Th17 inflammation contributes to persisting symptoms after treatment. Therefore, new treatment strategies involving Th17 downregulation are of interest. Several drugs that contain IL-17A inhibitors are increasingly used to inhibit Th17-driven inflammation in psoriasis^26^, although the CCR6-CCL20 axis is considered to be a pharmaceutical target also in several other inflammatory conditions^27^. Speculatively, Th17 inhibition could be a possible treatment option also for patients with LNB, by improving the convalescence of patients with symptoms that persist after treatment.

In addition to IL-17A, we found increased levels of the Th1-associated chemokine CXCL10 in the CSF of patients with LNB at baseline, in accordance with the results reported in other studies for both adults^4,7,22,28,29^ and in children^30,31^, and the levels were significantly reduced at follow-up. However, we did not find any association between CXCL10 and disease course, as has been reported in other studies^7,29^. Indeed, in our small study, we identified that the two patients who had the highest levels of CXCL10 post-treatment showed a high degree of inflammation, as evidenced by higher CSF cell counts, higher albumin ratio, and increased total IgG index. In addition, one of these patients had a longer period of convalescence (> 12 months). Thus, it is possible that a larger study would link Th1-associated inflammation with disease course, while in the present study with a smaller sample size, Th17-associated inflammation emerged as having the strongest association with the course of LNB.

Regarding the Th2-related chemokine CCL22, higher CSF levels were found in the CSF samples of patients, as compared with the controls, in accordance with previous findings^7,31^. However, we did not detect any associations between CCL22 and disease course. CCL22 is primarily produced by macrophages that are polarized towards an anti-inflammatory response^9^. CCL22 may have dual roles in LNB; acting as an anti-inflammatory agent that stimulates different mechanisms of healing^32^ and as an inflammatory mediator, as seen in EAE^9^. Thus, the relationship between CCL22 and clinical outcome in patients with LNB is still unclear.

Our study confirms previous findings of increased levels of the B cell-associated cytokines APRIL and BAFF in the CSF samples of patients with LNB^13,14^, although we could not relate these cytokine levels to clinical disease course, as shown previously in a retrospective cohort of patients with LNB^13^. It is possible that the association is time-dependent, since our study had no follow-up visits between 1 and 6 months post-treatment. The CSF levels of APRIL were significant higher in the controls than in the patients with LNB, both at baseline and at follow-up. This is somewhat surprising, since the findings contradict the results of the analyses of APRIL levels in our previous study^13^, obtained using the same laboratory method. As a consequence, these results are difficult to interpret. The median age of the controls differed significantly between the present study and our previous study, which might explain the discrepant results. Interestingly, we observed significantly higher levels of BAFF in the patients with radiculitis, possibly reflecting a higher B-cell activity associated with this symptom, as has also been shown by Ogrinc et al.^28^.

As many previous studies have highlighted before, the chemokine CXCL13 remains a reliable biomarker for differentiating LNB from many other neuroinflammatory conditions^14^. However, we could not relate CXCL13 to disease course, possibly indicating the impacts of other types of immune responses in the later stages of LNB. Senel et al.^33^ studied CXCL13 in the CSF samples of patients with LNB both before and after treatment; in line with our findings, the authors could not relate CXCL13 to disease course. In a recent study of 26 patients with bacterial or viral infections of the CNS^34^, not including LNB, increased levels of CXCL13 in the CSF samples of patients with a complicated disease course were observed. Although CXCL13 is a well-studied chemokine in the CSF of patients with different neuroinfectious and neuroinflammatory conditions, its potential role as a prognostic marker in patients with LNB needs to be studied further.

A limitation of the present study is the limited number of included patients. Nonetheless, the study is prospective and includes a follow-up LP after 1-month, as well as a standardized protocol for clinical follow-up during 1 year. Since cytokines and chemokines were not analyzed in serum samples, we cannot determine whether the increased levels in the CSF were the result of a systemic inflammation rather than a localized neuroinflammation, especially in the patients with a disrupted blood–brain barrier. However, several studies have clearly demonstrated compartmentalization of the inflammatory response to the CNS in patients with LNB, and almost no systemic inflammation has been observed in these patients^7,13,20,21,23,29,30,33^. Thus, the recorded inflammation in the CSF is very likely the result of an intrathecal inflammatory process. Additional strengths of this study are; the inclusion of a control group consisting of relatively healthy patients; investigations conducted with several PCR measurements, in order to avoid interference from other tick-borne infections; and the possibility to use the sensitive PEA methodology, which allowed for robust detection of the low-abundance proteins IL-17A and CCL20.

To summarize, in this prospective follow-up study of patients with LNB, we show that slow recovery after antibiotic treatment is associated with higher CSF levels of IL-17A and CCL20. These findings suggest IL-17A and CCL20 as prognostic markers and Th17-related immunity as a potential therapeutic target for patients with LNB, who show delayed post-treatment recovery due to a persisting intrathecal Th17-driven inflammation.

## Supplementary Information

Supplementary Information.

## Data Availability

Data and materials are available from the corresponding author upon request.

## Abbreviations

LNB: Lyme neuroborreliosis
*B*: *Borrelia*
s.l.: Sensu lato
CSF: Cerebrospinal fluid
Th: T helper
IFN: Interferon
IL: Interleukin
APRIL: A proliferation-inducing ligand
BAFF: B cell-activating factor
LP: Lumbar puncture
EFNS: European Federation of Neurological Societies
PEA: Proximity extension assay
NPX: Normalized Protein eXpression
EAE: Autoimmune encephalomyelitis
MS: Multiple sclerosis
