# Supplementary material for: Intrathecal Th17-driven inflammation is associated with prolonged post-treatment convalescence for patients with Lyme neuroborreliosis
Source: Sci Rep. 2023 Jun 15;13:9722. doi: 10.1038/s41598-023-36709-w (PMC10272195; doi:10.1038/s41598-023-36709-w)
Supplement: Supplementary file 1 — Supplementary Information. [file 41598_2023_36709_MOESM1_ESM.docx]

**Total nucleic acid extraction and cDNA synthesis from CSF and serum samples**

Total nucleic acids (NA) were extracted from 200 µL of CSF and 200 µL of serum using the MagNA Pure LC 2.0 Instrument (Life Science, Roche), and the MagNA Pure LC Total Nucleic Acid Isolation Kit (Life Science, Roche) using a 100-µL elution volume, according to the manufacturer’s instructions. Each extraction batch of 32 samples consisted of 30 samples, one positive control, and one negative control. As a positive control, 20 µL of PBS solution (pH 7.2) that contained a known number of *Borrelia (B.) afzelii* (strain Lu81) cells (ranging from 1 to 10^5^ cells per 20 μl) were spiked into 180 µL CSF (obtained from routine diagnostic section at the Department of Clinical Microbiology, County Hospital, Jönköping, Sweden). The strain of *B. afzelii* used was kindly provided by Professor Sven Bergström, Umeå University, Umeå, Sweden, and cultivated at 35°C for 8 days in Barbour-Stoenner-Kelly II medium with 6% rabbit serum, as described earlier, and counted using a phase-contrast microscope [1]. RNase-free water was used as a negative control. The NA were reverse-transcribed to cDNA using the illustra™ Ready-to-Go RT-PCR Beads kit (GE Healthcare Life Sciences, Little Chalfont, Buckinghamshire, UK). Fifteen microlitres of NA and 10 µL pd(N)6 random hexamer primers (0.25 µg/µL) were incubated for 5 min at 97°C and then mixed with one RT-PCR bead dissolved in 25 µL RNase-free water. The mixture was incubated for 30 min at 42°C, followed by 5 min at 97°C, producing 50 µL of cDNA.

All PCR analyses were performed at the Department of Biomedical and Clinical Sciences, Division of Inflammation and Infection, Linköping University, Linköping, Sweden.

**Detection of *Borrelia* spp. in CSF and serum samples**

Detection of *Borrelia* spp. (including *B. miyamotoi*) was performed using a genus-specific TaqMan real-time PCR assay. An in-house, genus-specific *Borrelia* *16S*-rDNA qPCR assay was performed using the LightCycler 480 real-time PCR System (Roche). The optimized mixture for PCR contained: Maxima Probe qPCR Mix; 200 nM *Borrelia*-F primer (5'-GCT GAG TCA CGA AAG CGT AG-3'); 200 nM *Borrelia*-R primer (5'-CAC TTA ACA CGT TAG CTT CGG TA-3'); 200 nM *Borrelia*-P probe (5'-FAM-CGC TGT AAA CGA TGC ACA CTT GGT-MGB-3'); 2 µl of template DNA; and RNase-free water added to give a final volume of 20 µl. The amplification conditions were: 5 min at 95°C, followed by 50 cycles of 95°C for 10 s, and then 60°C for 60 s. The primers and probe were designed *in silico* to detect all known *Borrelia* species. The specificity of the assay was confirmed using DNA from ten *B. burgdorferi* sensu lato (s.l.) genospecies and seven *Borrelia* relapsing fever genospecies, all of which tested positive. In addition, DNA from species from other genera of spirochetes (i.e., *Treponema* sp. and *Leptospira* sp.), as well as human DNA, tested negative in the newly designed assay. The sensitivity of the qPCR assay was confirmed in the laboratory using DNA extracted from a known number of *B. afzelii* (strain ACA-1) bacteria spiked in 200 µl of cerebrospinal fluid or synovial fluid, from which we could readily detect as few as 10 bacteria per 200 µl of either fluid. Using the above-described qPCR assay, we retrospectively screened the CSF samples for *Borrelia* spp. The primers *Borrelia*_F and *Borrelia*_R, and the probe *Borrelia*_P were designed to target the *Borrelia* spp. *16S* rRNA gene, to amplify a 116-bp long amplicon (Supplementary table S1). Positive samples were re-analyzed using a species-specific TaqMan real-time PCR assay, as previously described [2]. The primers Bm_F and Bm_R, and the probe Bm_P are designed to target the *B. miyamotoi* flagellin B gene (*flaB*) to amplify a 156-bp long amplicon (Supplementary table S1). To determine *B. burgdorferi* s.l. species of the samples positive in the real-time PCR assay, a nested, conventional PCR assay using primers targeting the intergenic spacer region between *5S* and *23S* rRNA genes, was applied as previously described [3, 4]. Nucleotide sequencing of the PCR products amplified by the conventional PCR assay to determine species of *Borrelia* was performed by Eurofins GATC Biotech (Konstanz, Germany). All sequences were confirmed by sequencing both strands. The obtained chromatograms were initially edited and analyzed using BioEdit Software v7.0 (Tom Hall, Ibis Therapeutics, Carlsbad, CA), and the sequences were examined using Basic Local Alignment Tool.

**Detection of *Anaplasma phagocytophilum* in CSF and serum samples**

Detection of *Anaplasma (A.)phagocytophilum* was performed using a TaqMan real-time PCR assay, as previously described [5]. The primers ApF and ApR and the probe ApM are designed to target the *A. phagocytophilum* citrate synthase gene (gltA), to amplify a 64-bp long amplicon (Supplementary table S1). As a positive control, a synthetic plasmid containing the target sequence of the TaqMan real-time PCR assay was used. The plasmid contained the target sequence, spanning nucleotides 304–420 of the *A. phagocytophilum gltA* gene (GenBank: AF304137), synthesised and cloned into the pUC57 vector (GenScript Inc., Piscataway, NJ, USA).

**Detection of *Rickettsia* spp. in CSF and serum samples**

Detection of *Rickettsia* spp. was performed using a TaqMan real-time PCR assay, as previously described [6]. The primers CS-F and CS-R, and probe CS-P are designed to target the *Rickettsia* spp. citrate synthase gene (gltA), to amplify a 74-bp long amplicon (Supplementary table S1). As a PCR-positive control, a synthetic plasmid containing the target sequence of the TaqMan real-time PCR assay was used. The plasmid contained the target sequence, spanning nucleotides 1102–1231 of the *R. rickettsii gltA* gene (GenBank: 59729), synthesised and cloned into the pUC57 vector (GenScript).

**Detection of *Neoehrlichia mikurensis* in CSF and serum samples**

Detection of *Neoehrlichia (N.) mikurensis* cDNA was performed using a SYBR Green real-time PCR assay, as previously described [7]. The primers Neo_*16S*_F and Neo_*16S*_R are designed to target the *N. mikurensis* *16S* rRNA gene, to amplify a 107-bp long amplicon (Supplementary table S1). As a positive control, cDNA samples positive for *N. mikurensis*, as confirmed by sequencing in an earlier study [7], were used in each run. Confirmatory PCR (using the GroEL target gene) of one positive serum sample was conducted at the Department of Clinical Microbiology, Sahlgrenska University Hospital, Gothenburg, Sweden, as previously described [8].

**Detection of tick-borne encephalitis virus in CSF and serum samples**

Detection of tick-borne encephalitis virus (TBEV) was performed using a duplex TaqMan real-time PCR assay, as previously described [9]. The primers and probes are designed to target all three subtypes of TBEV, to amplify a 68-bp and an 88-bp long amplicon, respectively (Supplementary table S1).

**Detection of *Babesia* spp. in CSF and serum samples**

Detection of *Babesia* spp. was performed using a SYBR Green real-time PCR assay, as previously described [10]. Primers BJ1 and BN2 are designed to target the *Babesia* spp. *18S* rRNA gene, to amplify a 411–452 bp long amplicon depending on the species of *Babesia* (Supplementary table S1). As a PCR-positive control, a synthetic plasmid containing the target sequence of the SYBR Green real-time PCR assay was used. The plasmid contained the target sequence, spanning nucleotides 467–955 of the *B. divergens* *18S* rRNA gene (GenBank: J439713), synthesized and cloned into the pUC57 vector (GenScript).

**Supplementary table S1.** Oligonucleotide primers and probes used for the molecular analysis of tick-borne pathogens.

| **Organism** | **Target gene** | **Primer/Probe name** | **Nucleotide sequence (5'→3')** | **Amplicon length (bp)** | **Reference** |
| --- | --- | --- | --- | --- | --- |
| *Borrelia* spp. | *16S* rRNA | *Borrelia*_F | GCT GAG TCA CGA AAG CGT AG | 116 | [11] |
|  |  | *Borrelia*_R | CAC TTA ACA CGT TAG CTT CGG TA |  |  |
|  |  | *Borrelia*_P | FAM-CGC TGT AAA CGA TGC ACA CTT GGT-MGB |  |  |
| *B. miyamotoi* | *flaB* | BM_F | AGA AGG TGC TCA AGC AG | 156 | [2] |
|  |  | BM_R | TCG ATC TTT GAA AGT GAC ATA T |  |  |
|  |  | BM_P | FAM-AGC ACA GGA GGG AGT TCA  AGC-BHQ1 |  |  |
| *A. phagocytophilum* | *gltA* | ApF | TTT TGG GCG CTG AAT ACG AT | 64 | [5] |
|  |  | ApR | TCT CGA GGG AAT GAT CTA ATA ACG T |  |  |
|  |  | ApM | FAM-TGC CTG AAC AAG TTA TG-BHQ1 |  |  |
| *Rickettsia* spp. | *gltA* | CS-F | TCG CAA ATG TTC ACG GTA CTT T | 74 | [6] |
|  |  | CS-R | TCG TGC ATT TCT TTC CAT TGT G |  |  |
|  |  | CS-P | FAM-TGC AAT AGC AAG AAC CGT AGG CTG GAT G-BHQ1 |  |  |
| *N. mikurensis* | *16S* rRNA | Neo_16S_F | GTA AAG GGC ATG TAG GCG GTT TAA | 107 | [7] |
|  |  | Neo_16S_R | TCC ACT ATC CTC TCT CGA TCT CTA GTT TAA |  |  |
|  | *groEL* | Neo-F | CGG AAA TAA CAA AAG ATG GA | 169 | [8] |
|  |  | Neo-R | ACC TCC TCG ATT ACT TTA G |  |  |
|  |  | Neo-probe | 6FAM-TTG GTG ATG GAA CTA CA-MGB |  |  |
| TBEV | 11,054–11,121^a^ | F-TBE 1 | GGG CGG TTC TTG TTC TCC | 68 | [12] |
|  |  | R-TBE 1 | ACA CAT CAC CTC CTT GTC AGA CT |  |  |
|  |  | TBE-probe-WT | FAM-TGA GCC ACC ATC ACC CAG ACA CA-BHQ1 |  |  |
|  | 1329–1416^a^ | TBEE-F6 | GGC TTG TGA GGC AAA GAA | 88 | [13] |
|  |  | TBEE-R2 | TCC CGT GTG TGG TTC GAC TT |  |  |
|  |  | TBEE-P4 | HEX-AAG CCA CAG GAC ATG TGT ACG CC-BHQ1 |  |  |
| *Babesia* spp. | *18S* rRNA | BJ1 | GTC TTG TAA TTG GAA TGA TGG | 411–452 | [10] |
|  |  | BN2 | TAG TTT ATG GTT AGG ACT ACG |  |  |

*B., Borrelia; A., Anaplsma*; TBEV, tick-borne encephalitis virus; ^a^Nucleotide fragment of the genome of TBEV strain Neudoerfl (U27495).

**Supplementary table S2.** Study protocol used at all visits.

|  |  | **At baseline** | **Follow up**  **1 month** | **Follow up**  **6 months** | **Follow up**  **12 months** |
| --- | --- | --- | --- | --- | --- |
| **Anamnesis** |  | **Duration (weeks)** | **Yes/No** | **Yes/No** | **Yes/No** |
| Radiculitis |  |  |  |  |  |
| Neck pain |  |  |  |  |  |
| Myalgia -and/or arthralgia |  |  |  |  |  |
| Fatigue |  |  |  |  |  |
| Headache |  |  |  |  |  |
| Facial nerve palsy |  |  |  |  |  |
| Fever (>38 ^o^C) |  |  |  |  |  |
| Vertigo |  |  |  |  |  |
| Concentration difficulties |  |  |  |  |  |
|  |  |  |  |  |  |
| **Neurological status** |  | **Normal/abnormal** | **Normal/abnormal** | **Normal/abnormal** | **Normal/abnormal** |
| Neck stiffness |  |  |  |  |  |
| Finger - nose |  |  |  |  |  |
| Romberg |  |  |  |  |  |
| Grasset |  |  |  |  |  |
| Toe - heel |  |  |  |  |  |
| Pupils |  |  |  |  |  |
| Eye movement |  |  |  |  |  |
| Nystagmus |  |  |  |  |  |
| Cranial nerves |  |  |  |  |  |
| Sensibility |  |  |  |  |  |
| Reflexes |  |  |  |  |  |
| Babinski sign |  |  |  |  |  |

**References**

1. Barbour AG. Isolation and cultivation of Lyme disease spirochetes. Yale J Biol Med. 1984 Jul-Aug;57(4):521-5.

2. Hovius JW, de Wever B, Sohne M, Brouwer MC, Coumou J, Wagemakers A, et al. A case of meningoencephalitis by the relapsing fever spirochaete Borrelia miyamotoi in Europe. Lancet. 2013 Aug 17;382(9892):658. doi: 10.1016/s0140-6736(13)61644-x.

3. Postic D, Assous MV, Grimont PA, Baranton G. Diversity of Borrelia burgdorferi sensu lato evidenced by restriction fragment length polymorphism of rrf (5S)-rrl (23S) intergenic spacer amplicons. Int J Syst Bacteriol. 1994 Oct;44(4):743-52. doi: 10.1099/00207713-44-4-743.

4. Wilhelmsson P, Fryland L, Borjesson S, Nordgren J, Bergstrom S, Ernerudh J, et al. Prevalence and diversity of Borrelia species in ticks that have bitten humans in Sweden. J Clin Microbiol. 2010 Nov;48(11):4169-76. doi: 10.1128/jcm.01061-10.

5. Henningsson AJ, Hvidsten D, Kristiansen BE, Matussek A, Stuen S, Jenkins A. Detection of Anaplasma phagocytophilum in Ixodes ricinus ticks from Norway using a realtime PCR assay targeting the Anaplasma citrate synthase gene gltA. BMC Microbiol. 2015 Aug 1;15:153. doi: 10.1186/s12866-015-0486-5.

6. Stenos J, Graves SR, Unsworth NB. A highly sensitive and specific real-time PCR assay for the detection of spotted fever and typhus group Rickettsiae. Am J Trop Med Hyg. 2005 Dec;73(6):1083-5.

7. Labbe Sandelin L, Tolf C, Larsson S, Wilhelmsson P, Salaneck E, Jaenson TG, et al. Candidatus Neoehrlichia mikurensis in Ticks from Migrating Birds in Sweden. PLoS One. 2015;10(7):e0133250. doi: 10.1371/journal.pone.0133250.

8. Grankvist A, Sandelin LL, Andersson J, Fryland L, Wilhelmsson P, Lindgren PE, et al. Infections with Candidatus Neoehrlichia mikurensis and Cytokine Responses in 2 Persons Bitten by Ticks, Sweden. Emerg Infect Dis. 2015 Aug;21(8):1462-5. doi: 10.3201/eid2108.150060.

9. Lindblom P, Wilhelmsson P, Fryland L, Sjowall J, Haglund M, Matussek A, et al. Tick-borne encephalitis virus in ticks detached from humans and follow-up of serological and clinical response. Ticks Tick Borne Dis. 2014 Feb;5(1):21-8. doi: 10.1016/j.ttbdis.2013.07.009.

10. Wilhelmsson P, Lövmar M, Krogfelt KA, Nielsen HV, Forsberg P, Lindgren PE. Clinical/serological outcome in humans bitten by Babesia species positive Ixodes ricinus ticks in Sweden and on the Åland Islands. Ticks Tick Borne Dis. 2020 Jul;11(4):101455. doi: 10.1016/j.ttbdis.2020.101455.

11. Gyllemark P, Wilhelmsson P, Elm C, Hoornstra D, Hovius JW, Johansson M, et al. Are other tick-borne infections overlooked in patients investigated for Lyme neuroborreliosis? A large retrospective study from South-eastern Sweden. Ticks Tick Borne Dis. 2021 Sep;12(5):101759. doi: 10.1016/j.ttbdis.2021.101759.

12. Schwaiger M, Cassinotti P. Development of a quantitative real-time RT-PCR assay with internal control for the laboratory detection of tick borne encephalitis virus (TBEV) RNA. J Clin Virol. 2003 Jul;27(2):136-45.

13. Gaumann R, Muhlemann K, Strasser M, Beuret CM. High-throughput procedure for tick surveys of tick-borne encephalitis virus and its application in a national surveillance study in Switzerland. Appl Environ Microbiol. 2010 Jul;76(13):4241-9. doi: 10.1128/aem.00391-10.
